# Retrospective Urine Metabolomics of Clinical Toxicology Samples Reveals Features Associated with Cocaine Exposure

**DOI:** 10.3390/metabo15090563

**Published:** 2025-08-22

**Authors:** Rachel K. Vanderschelden, Reya Kundu, Delaney Morrow, Simmi Patel, Kenichi Tamama

**Affiliations:** 1 Department of Pathology, University of Pittsburgh School of Medicine, Pittsburgh, PA 15261, USApatelsj@upmc.edu (S.P.); 2 Clinical Laboratories, University of Pittsburgh Medical Center Presbyterian Hospital, Pittsburgh, PA 15213, USA; 3 Department of Biological Sciences, University of Pittsburgh Kenneth P. Dietrich School of Arts and Sciences, Pittsburgh, PA 15260, USA; shk143@pitt.edu; 4 McGowan Institute for Regenerative Medicine, University of Pittsburgh, Pittsburgh, PA 15219, USA

**Keywords:** cocaine, cocaine-related disorders, urine drug screening, metabolomics, biomarkers, liquid chromatography–mass spectrometry

## Abstract

**Background/Objectives:** Cocaine is a widely used illicit stimulant with significant toxicity. Despite its clinical relevance, the broader metabolic alterations associated with cocaine use remain incompletely characterized. This study aims to identify novel biomarkers for cocaine exposure by applying untargeted metabolomics to retrospective urine drug screening data. **Methods:** We conducted a retrospective analysis of a raw mass spectrometry (MS) dataset from urine comprehensive drug screening (UCDS) from 363 patients at the University of Pittsburgh Medical Center Clinical Toxicology Laboratory. The liquid chromatography–quadrupole time-of-flight mass spectrometry (LC-qToF-MS) data were preprocessed with MS-DIAL and subjected to multiple statistical analyses to identify features significantly associated with cocaine-enzyme immunoassay (EIA) results. Significant features were further evaluated using MS-FINDER for feature annotation. **Results:** Among 14,883 features, 262 were significantly associated with cocaine-EIA results. A subset of 37 more significant features, including known cocaine metabolites and impurities, nicotine metabolites, norfentanyl, and a tryptophan-related metabolite (3-hydroxy-tryptophan), was annotated. Cluster analysis revealed co-varying features, including parent compounds, metabolites, and related ion species. **Conclusions:** Features associated with cocaine exposure, including previously underrecognized cocaine metabolites and impurities, co-exposure markers, and alterations in an endogenous metabolic pathway, were identified. Notably, norfentanyl was found to be significantly associated with cocaine -EIA, reflecting current trends in illicit drug use. This study highlights the potential of repurposing real-world clinical toxicology data for biomarker discovery, providing a valuable approach to identifying exposure biomarkers and expanding our understanding of drug-induced metabolic disturbances in clinical toxicology. Further validation and exploration using complementary analytical platforms are warranted.

## 1. Introduction

Cocaine is one of the most widely used illicit stimulants globally, associated with diverse toxic effects, including cardiovascular complications, neuropsychiatric syndromes, and long-term addiction [1,2,3,4,5]. Despite its clinical and forensic relevance, the broader metabolic alterations associated with cocaine use remain incompletely understood, especially in real-world clinical settings. Routine urine drug screening relies on immunoassays and subsequent mass spectrometry-based assays to detect known drugs and their primary metabolites [6]. These approaches are practical for confirming exposure but do not capture the full extent of biochemical changes induced by drug use.

Metabolomics, the global profiling of small molecules in biological systems, enables more profound insight into drug-induced metabolic perturbations. Untargeted metabolomics has been increasingly applied in toxicology research to identify novel biomarkers, uncover altered pathways, and better understand drug mechanisms [7,8,9,10,11,12,13]. With advances in liquid chromatography–high-resolution mass spectrometry (LC-HRMS), especially liquid chromatography–quadrupole time-of-flight mass spectrometry (LC-qToF-MS), untargeted data acquisition is now routinely incorporated into clinical toxicology workflows, creating new opportunities for retrospective feature-level analysis [6].

As part of routine clinical practice, we conduct urine comprehensive drug screening (UCDS) (>500 cases per month) to identify causative drugs and chemicals for intoxication and to monitor the compliance of prescription drugs at the University of Pittsburgh Medical Center Clinical Toxicology Laboratory. In urine comprehensive drug screening, we acquire mass spectral data of analytes in an untargeted manner using LC-qToF-MS with all ion fragmentation scans [6,14]. LC-qToF-MS reveals 5000–10,000 features per specimen, but our clinical practice focuses only on 50 features of xenobiotics (drugs and their metabolites) per sample at best, leaving the remaining features unannotated and unanalyzed. These unannotated features represent an underutilized resource that may harbor clinically actionable biomarkers and provide novel insights into the metabolic effects of drug exposure.

In this study, we repurposed data from our UCDS, which employs LC-qToF-MS for qualitative toxicological assessment. Although the primary goal of this platform is drug screening, the untargeted data generated by LC-qToF-MS are suitable for downstream metabolomics. Using an archived mass spectrometry dataset, we conducted a retrospective metabolomics study, classifying specimens using qualitative cocaine -enzyme immunoassay (EIA) results as metadata. By comparing the metabolic profiles of EIA-positive and EIA-negative samples, we aimed to identify discriminating features associated with cocaine use.

While cocaine itself is not a novel analyte, our approach demonstrates the value of mining routine clinical toxicology data for metabolomic insights. Specifically, we show how retrospective feature-level analysis can reveal potential biomarkers of cocaine exposure, identify co-eluting compounds and metabolites, and shed light on broader biochemical alterations. We also address analytical challenges, including annotating isomeric features and possible in-source fragmentation, which are common in LC-MS-based metabolomics [15,16,17,18,19].

This study highlights the translational potential of integrating metabolomics into clinical toxicology workflows. By leveraging existing diagnostic data, we provide a model for low-barrier biomarker discovery and a deeper understanding of drug-induced metabolic profiles using real-world clinical samples.

## 2. Materials and Methods

### 2.1. Specimens

The UCDS datasets were originally derived from the urine specimens collected from 363 patients (242 patients with an age of ≥20 years and 121 patients with an age of ≤19 years old) between March and May 2021 and in June 2022 (Table 1). The specimens were sent from emergency departments of UPMC hospitals and clinics in Pittsburgh, PA. These specimens underwent an initial EMIT-II-based qualitative drug screening panel, including the one for cocaine (cocaine-EIA) with a cut-off of 300 ng/mL for benzoylecgonine (Siemens Healthineers USA, Malvern, PA, USA). Mass spectrometry-based analysis was performed within four hours of sample receipt at the clinical toxicology laboratory. One volume of the urine specimen was mixed with four volumes of distilled water spiked with nitrazepam and tenoxicam as internal standards (40 ng/mL final). A 3 μL aliquot of the diluted specimen was then directly injected into the LC-qToF-MS system.

### 2.2. LC-qToF-MS Conditions/Settings

A Xevo^®^ G2-S QToF mass spectrometer and an ACQUITY UPLC^®^ I-Class system with an UPLC HSS C18 column (Waters, Milford, MA, USA) were used for UCDS. The acquired mass spectrometry datasets were analyzed using the UNIFI^®^ Scientific Information System (Waters, Milford, MA, USA) to screen compounds via retention time, monoisotopic mass accuracy, and fragment ions matching in the UCDS.

Gradient elution chromatography in reverse-phase systems was achieved using the mobile Phase A (5 mM ammonium formate, pH 3.0) and mobile Phase B (Acetonitrile containing 0.1% formic acid). The gradient elution was performed as follows in the 15 min run: the column was equilibrated with 87% A for 0.5 min, then the concentration of A was decreased to 50% over 9.5 min, then decreased to 5% over 0.75 min and held for 1.5 min, then increased back to 87% over 2 min. The column temperature was set at 50.0 °C.

Electron spray ionization (ESI) in the positive ionization mode was used. The ESI settings were as follows: capillary, 0.80 kV; sample cone voltage, 25 V; source temperature, 150 °C; desolvent gas, 800 L/h at 400 °C; and cone gas, 20 L/h. MS data of both precursor and product ions in a 50–1000 Da mass range were collected in a non-targeted manner using MSE technology with the following settings: collision cell setting, low energy 6 eV; high energy ramp, 10 to 40 eV; scan time, 0.1 sec; data format, continuum).

### 2.3. Data Export and Preprocessing

**Figure 1:**
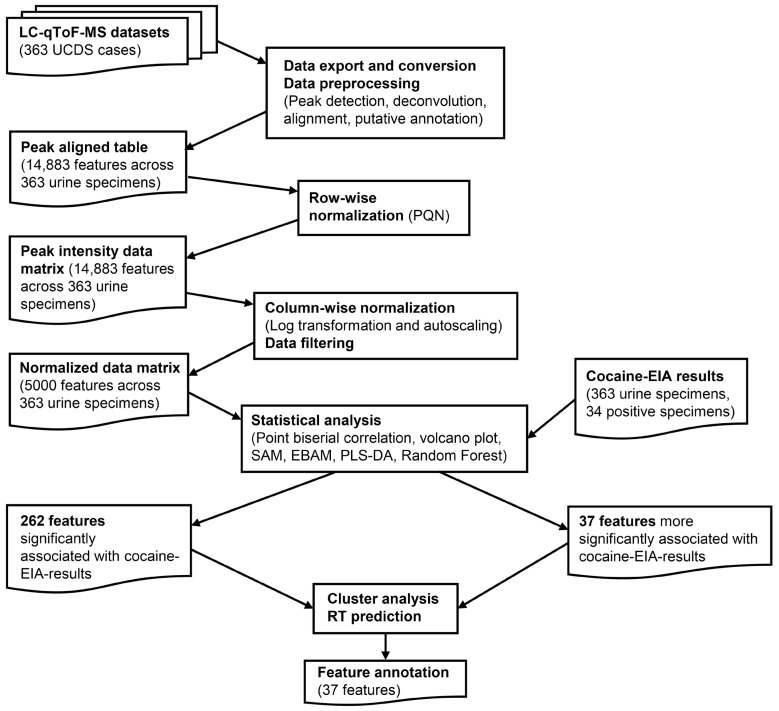
Analytical and computational workflow for untargeted urine metabolomics analysis of cocaine-associated features.

An overview of the overall data processing and analysis workflow, from raw mass spectrometry data export through to statistical analysis, is presented in Figure 1. MS datasets in .uep files were first exported from UNIFI as .raw files. After removing any personal identifiers and lock mass data, these files were converted to mzML files in the centroid mode using ProteoWizard msConvert [20].

From the MS datasets (.mzML), peak detection, deconvolution, alignment, and putative annotation of the aligned features were performed using MS-DIAL version 4.90 [21], resulting in a peak aligned table comprising 14,883 features across 363 urine specimens. As for the MS libraries, 48,189 MS records (both experimental and predicted) of human urine metabolites were downloaded from Human Metabolome Database Ver. 5 [22], converted into the NIST msp format library, and added to the existing 56,694 MS records in MS libraries (MassBank, MassBank EU, ReSpect, GNPS, Fiehn HILIC, CASMI2016 MetaboBASE, RIKEN PlaSMA authentic standards, RIKEN PlaSMA bio-MS/MS (MSI level 1, 2, 3, or 4) from plant tissues, Karolinska institute and Gunma (GIAR) zic-HILIC deconvoluted MS2 spectra in data-independent acquisition, Fiehn Pathogen Box, and the Fiehn/Vaniya natural product library) available for MS-DIAL. Mass accuracy parameters for feature detection and spectral deconvolution were set to 0.01 Da for MS1 and 0.05 Da for MS2. For MS/MS spectral annotation, the accurate mass tolerances were set to 0.025 Da for MS1 and 0.25 Da for MS2, reflecting the modest mass accuracy due to the absence of lock mass correction. Peak detection was performed with a minimum peak height of 5000 amplitude and a mass slice width of 0.1 Da. For peak alignment, the retention time tolerance was set to 0.25 min, and the MS1 tolerance was 0.025 Da. From the peak-aligned table generated by MS-DIAL, the peak intensity data matrix was constructed by applying row-wise normalization using probabilistic quotient normalization (PQN). The median of the entire dataset was used as the reference to minimize the effects of varying urine concentrations and improve comparability across specimens [23].

### 2.4. Data Analysis

The peak intensity data matrix was further processed for data exploration and analysis using MetaboAnalyst (both the web version and the R version or MetaboAnalystR), a unified platform developed for comprehensive metabolomic data analysis [24,25,26,27,28]. Statistical analyses were selected to balance sensitivity for feature discovery (e.g., volcano plots) with robustness to complex, high-dimensional data and class imbalance (e.g., Random Forest), ensuring comprehensive identification and validation of cocaine-associated metabolic features. Both column-wise normalization and data filtering were first applied to the peak intensity data matrix to generate the normalized data matrix before statistical analyses were performed using MetaboAnalyst. Column-wise normalization with log transformation and autoscaling was performed to make each feature comparable. Data filtering was performed to remove non-informative features with very small or near-constant values, reducing the number of features to 5000, the maximum number of features that MetaboAnalyst can analyze. The cocaine-EIA results were used as metadata to categorize the specimens for further statistical analysis.

Point biserial correlation analysis was performed with MetaboAnalystR to explore the correlations between metabolomic features and EIA results (binary metadata). The significant features with FDR < 0.05 were selected.

A volcano plot was performed with MetaboAnalystR to combine the results from fold change analysis and t-tests. Fold changes are calculated as the ratios between two group means >2 using the original data before normalization. T-tests were also conducted for each feature with a *p*-value threshold of <0.05.

Significance analysis of microarrays and metabolites (SAM) [29] and empirical Bayesian analysis of microarrays and metabolites (EBAM) [30] were performed with MetaboAnalystR to select the significant features on the basis of the false discovery rate (FDR). The SAM plot is a scatter plot of the observed relative difference versus the expected relative difference estimated by data permutation. The EBAM was performed to select significant features at a posterior delta of 0.9 and FDR < 0.05.

Partial least squares discriminant analysis (PLS-DA) was performed with MetaboAnalystR to select significant features to predict EIA results (metadata-based classification) by calculating the weighted sum of absolute regression coefficients for the overall components. Features were retained only if 2000-permutation testing yielded statistically significant results.

Random Forest was performed using R packages (caret (Ver. 6.0-94) and randomForest (Ver. 4.6-14)) after class imbalance of the samples was corrected by a combination of random oversampling of the non-dominant class samples and undersampling of the dominant class samples using the R package ROSE (Ver. 0.0-4).

Cluster analysis was performed by calculating the feature-to-feature Pearson correlation coefficients from the transposed peak intensity matrix of cocaine-associated features for the cocaine-EIA-positive specimens. The resulting correlation matrix was visualized as a heatmap using the pheatmap package in R, and hierarchical clustering was performed using Ward’s method (ward.D2). Features were grouped into seven clusters (Clusters A–G; Table 2A–G) by high-level dendrogram slicing (k = 7). Middle-level dendrogram slicing (k = 14) was applied to identify sub-clusters (e.g., A1, A2, A3), and low-level dendrogram slicing (k = 50) was performed to define terminal branch clusters (e.g., A1-1, A1-2, A1-3). These sub-clusters and terminal branches represent co-varying features that may reflect structurally related compounds or analytical associations.

### 2.5. Feature Annotations

MS-FINDER version 3.61 [31,32] was utilized to further elucidate the putative chemical structures of selected features on the basis of MS/MS fragmentation patterns and elemental composition. Minor metabolites and impurities of recreational drugs, including cocaine, tobacco, opioids, and benzodiazepines, and major novel psychoactive substances (NPS), were also fed into MS-FINDER through the user-defined database prepared manually. The SMILES and InChIKey of these metabolites were manually prepared using ChemDraw (Ver 23.1.2) (PerkinElmer, Waltham, MA, USA) if not available in the public database (Human Metabolome Database and PubChem).

MS1 mass spectra and extracted ion chromatograms (EICs) were visualized using MZmine 4.78 [33,34]. The retention times were also predicted using the R package Retip [35], which was trained using our retention time data of the compounds.

## 3. Results

**Figure 2:**
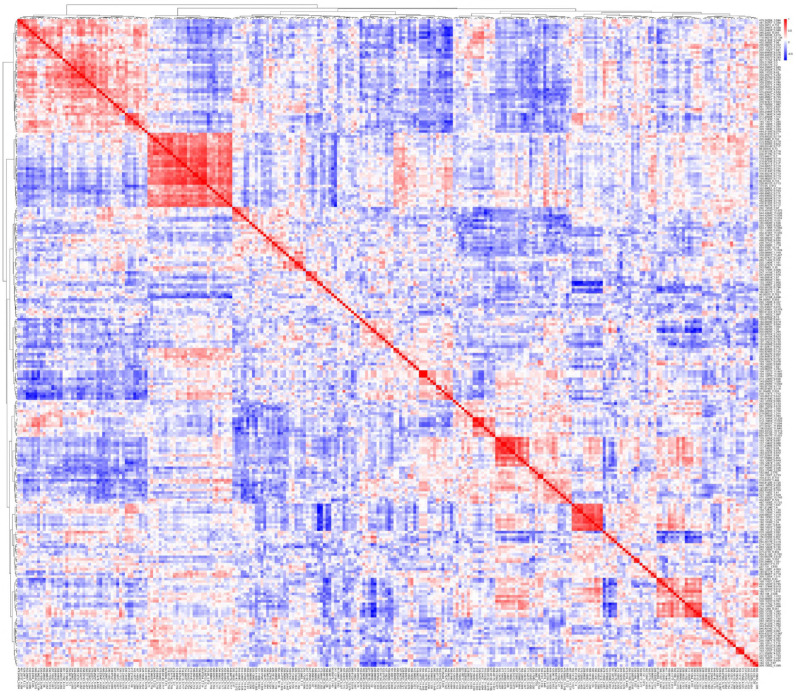
Correlation heatmap and hierarchical clustering of 262 cocaine-associated features. The heatmap displays pairwise Pearson correlation coefficients among the 262 cocaine-associated features. Both axes represent the same feature set, ordered identically according to hierarchical clustering. The color indicates correlation strength (red: positive; blue: negative). The accompanying dendrogram (top and left) was generated using Ward’s linkage on 1 − Pearson correlation distance. Block-like red regions along the diagonal reflect clusters of co-varying features, used to define Clusters A–G (Table 2A–G) and guide further interpretation of structurally related signals.

Among the 14,883 features in the dataset, 262 were initially identified as significantly associated with cocaine-EIA results by at least one statistical analysis. However, the initial putative annotation of these features using MS-DIAL and MS-FINDER met with only limited success, presumably because of the limited inclusion of minor cocaine metabolites, cocaine-related impurities, and their metabolites, including Phase II metabolites, in the public databases [36]. Thus, we created a user-defined database covering these analytes for MS-FINDER. In-source fragments might also be mistaken for precursor ions [15,37]. To better interpret these features, we performed cluster analyses of the 262 features in the normalized data matrix, based on the assumption that structurally related compounds—such as the parent drugs, their immediate metabolites, and associated ion signatures—would cluster together. The features were grouped into seven high-level clusters (Clusters A–G), corresponding to Table 2A–G. This hierarchical clustering approach was designed to reflect co-variation patterns and facilitate the interpretation of unannotated or partially annotated signals (Table 2 and Figure 2).

**Figure 3:**
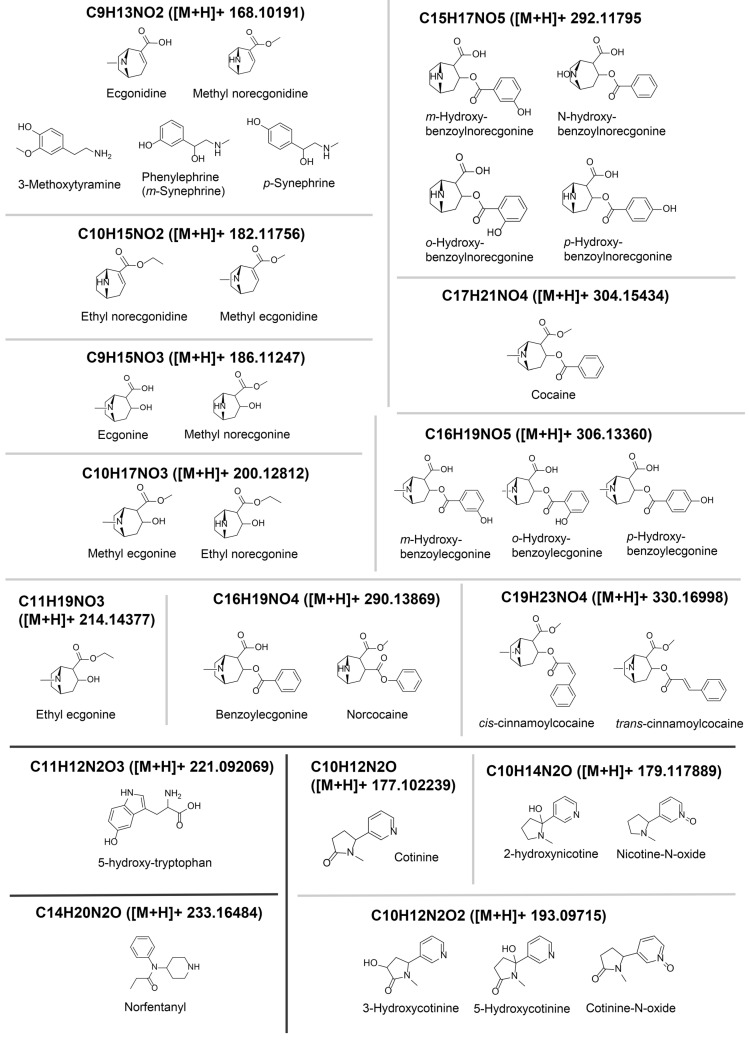
Chemical structures of the molecular candidates for annotated features significantly associated with the cocaine-EIA results. These molecules are grouped by molecular formula. Each structure was drawn using ChemDraw (Ver. 23.1.2) (PerkinElmer, Waltham, MA, USA).

On the basis of these analyses, we attempted to annotate 37 more significant features that were selected by more than one analysis and thus were more strongly associated with the cocaine-EIA results. These 37 significant features and their annotations are summarized in Table 3. These 37 features are classified as cocaine and its metabolites, cocaine impurities, nicotine and its metabolites, opioid (norfentanyl), and tryptophan/serotonine metabolite (3-hydroxy-tryptophan) (Table 3 and Figure 3).

### 3.1. Features 200.12935_0.795, 200.13182_1.007 and 200.13109_4.253

These features likely correspond to the molecular formula of C_10_H_17_NO_3_ ([M + H]+ 200.12812). Possible chemical structures for C_10_H_17_NO_3_ elucidated by MS-FINDER include ethyl norecgonine and methyl ecgonine among the cocaine metabolites (Figure 3), and methyl ecgonine is the top candidate for these three features. The RT of ethyl norecgonine is predicted to be slightly longer than that of methyl ecgonine (RT: 0.80 min, determined experimentally) (Table 4), and thus the feature 200.13182_1.007 is putatively annotated to be ethyl norecgonine. Even though the annotation of the feature 200.13109_4.253 is unclear, this feature is clustered in the low slicing group together with cocaine 304.15652_4.399 and its metabolites, including the feature 200.13182_1.007 (putative ethyl norecgonine) in Terminal Branch Cluster G1-4 in Cluster G (Table 2G), and this feature 200.13109_4.253 is likely another cocaine metabolite or an in-source fragment thereof.

### 3.2. Features 304.15652_4.399 and 304.16803_4.254

These features likely correspond to the molecular formula of C_17_H_21_NO_4_ ([M + H]+ 304.15434). Among the structures proposed by MS-FINDER, one of the top candidates includes cocaine (Figure 3), which has an RT of 4.46 min, consistent with these features. The feature 304.15652_4.399 is clustered in the low slicing group together with other cocaine metabolites such as the feature 200.13182_1.007 (putative ethyl norecgonine) in Terminal Branch Cluster G1-4 in Cluster G (Table 2G), as discussed previously, whereas the feature 304.16803_4.254 is clustered together in the low slicing group with an illicit cocaine impurity (cinnamoylcocaine, 330.17252_5.513) in Terminal Branch Cluster A2-1 and the middle slicing group with a cocaine metabolite (benzoylecgonine, 290.14951_3.314) in Sub-cluster A2 in Cluster A (Table 2A), as discussed below.

These two closely eluting features with almost the same *m*/*z* values are likely a representation of cocaine. Manual inspection of the extracted ion chromatograms (EICs) confirmed that these features share a single chromatographic peak shape with slightly offset apexes. Given the absence of lock mass correction, minute *m*/*z* drift and centroid rounding likely caused the algorithm to assign separate features to the same molecular ion.

### 3.3. Feature 182.123_0.87

This feature likely corresponds to the molecular formula of C_10_H_15_NO_2_ ([M + H]+ 182.11756). Possible chemical structures for C_10_H_15_NO_2_ elucidated by MS-FINDER include ethyl norecgonidine and methyl ecgonidine (anhydroecgonine methylester), among the cocaine metabolites (Figure 3). While methyl ecgonidine is the top match on the basis of structure scoring, its experimentally determined RT is 1.10 min, which does not match the observed RT of 0.87 min. Since ethyl norecgonidine is predicted to have an even longer RT than methyl ecgonidine (Table 4), it is also an unlikely match for this feature.

An alternative and more plausible explanation is that this feature represents an in-source loss of water ([M + H–H_2_O]+) from a parent ion at *m*/*z* 200.1291, corresponding to methyl ecgonine 200.12935_0.795. Although methyl ecgonine and its in-source fragment are expected to co-elute, they can be assigned slightly different retention times (0.80 min vs. 0.87 min) by MS-DIAL. This difference likely reflects the way MS-DIAL independently detects and deconvolutes each ion feature based on apex intensity within its respective *m*/*z* window, rather than a true chromatographic separation, in the peak alignment process [21]. Furthermore, this feature was observed in the same peak-slicing cluster as cocaine 304.15652_4.399 and other known cocaine-related metabolites in Terminal Branch Cluster G1-4 in Cluster G (Table 2G), supporting its biological relevance to cocaine metabolism.

### 3.4. Features 186.11491_0.834, 186.11212_1.025, and 186.11717_1.818

These features likely correspond to the molecular formula of C_9_H_15_NO_3_ ([M + H]+ 186.11247). Possible chemical structures for C_9_H_15_NO_3_ elucidated by MS-FINDER include ecgonine and methyl norecgonine among the cocaine metabolites (Figure 3). Ecgonine is the top candidate for these three features, whereas methyl norecgonine is one of the top hits for the feature (186.11212_1.025). The RT of methyl norecgonine is predicted to be longer than that of ecgonine (RT: 0.80 min, determined experimentally) (Table 4). Thus, ecgonine is likely the molecule of the feature 186.11491_0.834, and methyl norecgonine would be the molecule of the feature 186.11212_1.025.

**Figure 4:**
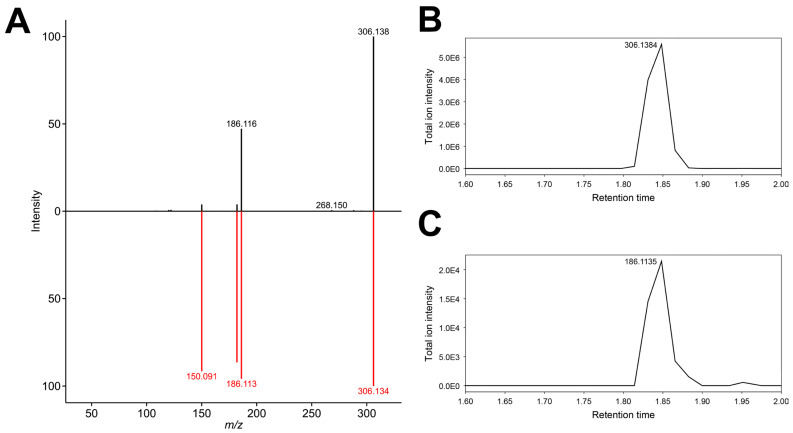
The deconvoluted MS/MS spectrum of the feature 306.14551_1.781 and in silico MS/MS spectrum matching of hydroxy-benzoylecgonine (**A**) and extracted ion chromatograms of *m*/*z* 306.1384 (**B**) and *m*/*z* 186.1135 (**C**) between 1.6 and 2.0 min. The striking similarity in the retention profiles between the ion pairs 306.1384/186.1135 (Panels **B**,**C**) suggests a possible analytical relationship, likely through in-source fragmentation of hydroxy-benzoylecgonine.

To seek an alternative explanation for the feature 186.11717_1.818, we manually reviewed the aligned peak table generated by MS-DIAL for other cocaine metabolites [8,38] eluting around 1.8 min and identified another feature (306.14551_1.781), for which hydroxy-benzoylecgonine (C_16_H_19_NO_5_, [M + H]+ 306.13360) is elucidated to be the top candidate by MS-FINDER (Figure 3). This feature was filtered out by MetaboAnalyst; therefore, it is not included in the normalized data matrix or cluster analysis. The MS2 spectrum of this feature includes an ion 186.1163, which can be generated after the neutral loss of C_7_H_4_O_2_, which corresponds to the hydroxybenzaldehyde moiety, leaving the ecgonine moiety (Figure 4A). The peak patterns of these features look similar to each other in the EIC of MS1 around 1.8 min (Figure 4B,C).

In-source fragmentation is a relatively common phenomenon in the ESI-based analysis of natural molecules [39]. Ion fragments generated through in-source fragmentation and a collision cell are comparable [40,41]. We speculate that the feature 186.11717_1.818 might be an in-source fragment of hydroxy-benzoylecgonine.

### 3.5. Features 330.17252_5.513 and 330.17572_5.651

These features likely correspond to the molecular formula of C_19_H_23_NO_4_ ([M + H]+ 330.16998). Possible chemical structures for C_19_H_23_NO_4_ elucidated by MS-FINDER include cis- and trans-cinnamoylcocaine (Figure 3), which are tropane alkaloids in coca leaves and impurities of illicit cocaine products [42,43].

### 3.6. Features 168.11021_0.847, 168.10132_1.248, and 168.10931_1.568

These features likely correspond to the molecular formula of C_9_H_13_NO_2_ ([M + H]+ 168.10191), which includes ecgonidine and methyl norecgonidine among the cocaine metabolites (Figure 3). The RT of methyl norecgonine is predicted to be slightly longer than that of ecgonidine (RT: 0.84 min, determined experimentally) (Table 4); thus, the feature 168.11021_0.847 would be annotated to be ecgonidine, whereas the features 168.10132_1.248 or 168.10931_1.568 would be methyl norecgonine.

Both features (168.10132_1.248 and 168.10931_1.568) are grouped together in the low slicing group with the feature 186.11212_1.025, which is suspected to be methyl norecgonine in Terminal Branch Cluster C2-1 in Cluster C (Table 2C) (see above). Notably, the mass difference between this feature and the 168 *m*/*z* features is approximately 18 Da, suggesting a possible in-source loss of water ([M + H–H_2_O]+) for methyl norecgonine. This raises the possibility that 186.11212_1.025 may be the intact parent ion, while 168.11021_0.847 and 168.10132_1.248 could include contributions from its in-source fragment of 186.11212_1.025.

Furthermore, this *m*/*z* 168 signal also matches several isomeric compounds unrelated to cocaine metabolism, including p-synephrine, phenylephrine (m-synephrine), and 3-methoxytyramine (Figure 3). Synephrine is occasionally reported as an adulterant in street cocaine and is also found in weight-loss supplements [44], while phenylephrine is a common over-the-counter decongestant. 3-Methoxytyramine, an immediate dopamine metabolite, is biologically relevant in the context of cocaine use, which alters dopaminergic signaling. Increased urinary levels of 3-methoxytyramine have been reported during early abstinence [45].

Given the physicochemical similarity with the shared formula, overlapping MS/MS spectra, and similar RTs (p- and m-synephrine for 0.83 min and 3-methoxytyramine for 0.98 min experimentally) and possible occurrence of in-source fragmentation, these features, especially 168.11021_0.847 and 168.10132_1.248, might represent composite signals from multiple co-eluting compounds and an in-source fragment of another ion.

### 3.7. Feature 214.15097_0.935

This feature likely corresponds to the molecular formula of C_11_H_19_NO_3_ ([M + H]+ 214.14377), for which MS-FINDER suggests ethyl ecgonine as a top candidate molecule among the cocaine metabolites (Figure 3). The RT of ethyl ecgonine is 0.93 min (experimentally determined), matching that of this feature. Thus, the feature 214.15097_0.935 is likely the representation of ethyl ecgonine.

### 3.8. Feature 233.17049_3.226

This feature likely corresponds to the molecular formula of C_14_H_20_N_2_O ([M + H]+ 233.16484). Possible chemical structures for C_14_H_20_N_2_O elucidated by MS-DIAL and MS-FINDER include norfentanyl (Figure 3), a dealkylated metabolite of fentanyl, but not any cocaine metabolites. The RT of norfentanyl is 3.25 min (experimentally determined), matching that of this feature. Thus, the feature 233.17049_3.226 is likely the representation of norfentanyl. Opioids such as fentanyl are often co-ingested with cocaine in the fourth wave of the opioid crisis [46,47], supporting this finding.

### 3.9. Feature 292.1264_4.051

This feature likely corresponds to the molecular formula of C_15_H_17_NO_5_ ([M + H]+ 292.11795), for which hydroxy-benzoylnorecgonine is suggested among the cocaine metabolites by MS-FINDER (Figure 3). There are four isomers possible, according to the hydroxylation site (*o*-, *m*-, *p*-, or N-), which was not discernible by the mass spectra in this case. MS-FINDER lists N-hydroxy-benzoylnorecgonine as the top molecule among the cocaine metabolites, but it also lists other isomers among the top metabolites as well. N-hydroxy-benzoylnorecgonine elutes at 3.74 min, and (*o*-, *m*-, or *p*-)hydroxy-benzoylnorecgonine elutes at 2.48 min in a similar but slightly shorter gradient RPLC program (10 min instead of 15 min in our program) [38]. Thus, N-hydroxy-benzoylnorecgonine would be the putative annotation for the feature 292.1264_4.051, but (*o*-, *m*-, or *p*-)hydroxy-benzoylnorecgonine cannot be ruled out as well.

### 3.10. Feature 290.14951_3.314

This feature likely corresponds to the molecular formula C_16_H_19_NO_4_ ([M + H]+ 290.13869), which matches both benzoylecgonine (experimentally observed RT: 2.90 min) and norcocaine (RT: 4.54 min) among the cocaine metabolites (Figure 3). According to the MS2 pattern and RT, this feature aligns more closely with benzoylecgonine. However, the RT is approximately 0.4 min longer than expected for benzoylecgonine.

The closer examination suggests that this feature may represent the tailing portion of a large benzoylecgonine peak (the feature 290.14487_2.854). Notably, this main peak was removed during data filtering by MetaboAnalyst, likely due to its detection across both cocaine-EIA-positive and -negative specimens, which is possibly due to its rather ubiquitous presence across both cocaine-EIA-positive and -negative specimens because of its relatively high cut-off level (300 ng/mL). Meanwhile, MS-DIAL seems to detect the tail of the large benzoylecgonine peak as a distinct feature, a known limitation of peak detection algorithms in the presence of broad or asymmetric chromatographic peaks [48,49].

### 3.11. Feature 221.08717_1.055

This feature likely corresponds to the molecular formula of C_11_H_12_N_2_O_3_ ([M + H]+ 221.092069). Possible chemical structures for C_11_H_12_N_2_O_3_ elucidated by MS-FINDER include 5-hydroxy-tryptophan (RT: 0.90 experimentally) (Figure 3), an immediate precursor of the neurotransmitter serotonin generated from tryptophan [50], but no cocaine metabolites are suggested by MS-FINDER. Cocaine use is associated with altered tryptophan and serotonin metabolism [51], with elevated plasma serotonin levels observed in abstinent cocaine users [52]. Thus, the feature 221.08717_1.055 is annotated as 5-hydroxy-tryptophan.

### 3.12. Features 193.10092_0.876 and 193.24315_0.872

These features likely correspond to the molecular formula of C_10_H_12_N_2_O_2_ ([M + H]+ 193.09715). Possible chemical structures for C_10_H_12_N_2_O_2_ elucidated by MS-FINDER include hydroxycotinines and cotinine N-oxide (Figure 3). Among these metabolites, 3-hydroxycotinine is the dominant nicotine metabolite in the urine [53]. The RT is experimentally determined to be 0.90 min, whereas that of cotinine N-oxide is 0.96 min. Thus, the feature 193.10092_0.876 is likely 3-hydroxycotinine, but minor contributions from other isomeric nicotine metabolites (e.g., 5-hydroxycotinine and cotinine-N-oxide) (Figure 3) cannot be ruled out.

**Figure 5:**
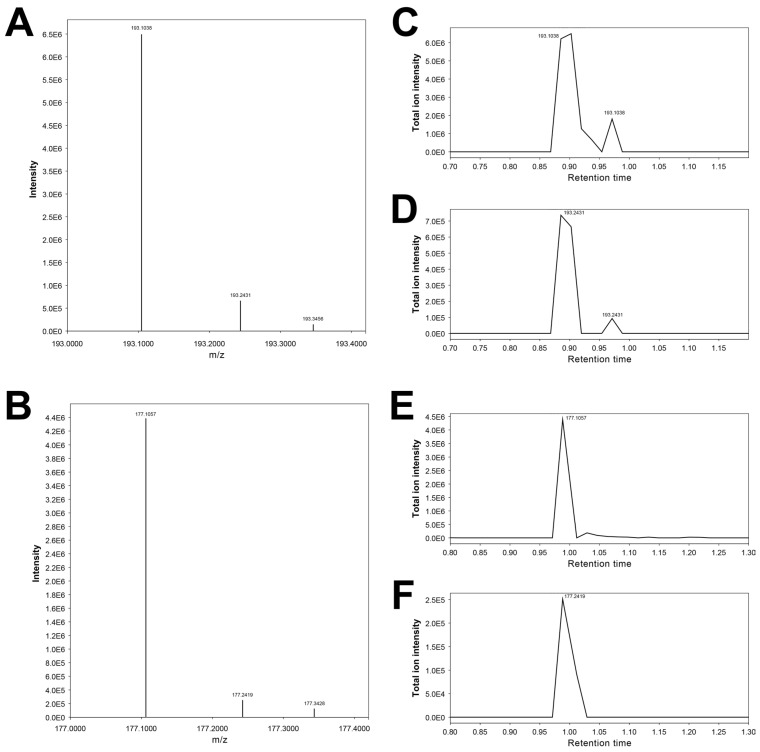
MS1 spectra and extracted ion chromatograms (EICs) from a urine specimen containing hydroxycotinine (**A**–**C**) and cotinine (**D**–**F**). Panels A and B show MS1 spectra acquired at retention times of 0.90 min and 0.98 min, respectively. Panels **C**,**D** display EICs for *m*/*z* 193.1038 and 193.2431, while Panels **E**,**F** show EICs for *m*/*z* 177.1057 and 177.2419. The striking similarity in retention profiles between the ion pairs 193.1038/193.2431 (Panels **C**,**D**) and 177.1057/177.2419 (Panels **E**,**F**) suggests a possible analytical relationship, although the exact mechanism remains undetermined. Co-elution and matching chromatographic shapes support this association.

**Figure 6:**
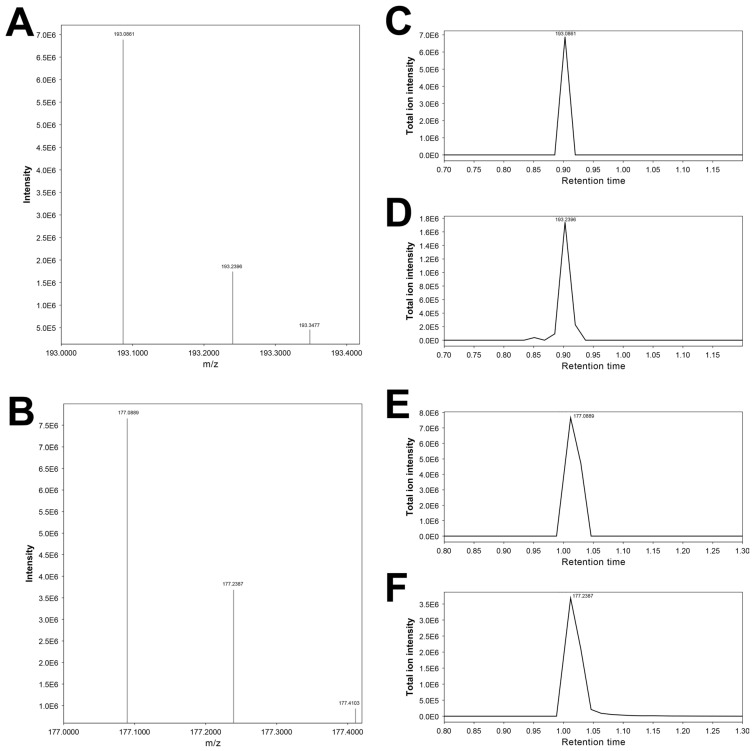
MS1 spectra and extracted ion chromatograms (EICs) from a blank urine sample spiked with 3-hydroxycotinine (**A**–**C**) and cotinine (**D**–**F**). Panels **A**,**B** show MS1 spectra acquired at retention times of 0.90 min and 1.01 min, corresponding to authentic 3-hydroxycotinine and cotinine, respectively. Panels **C**,**D** display EICs for *m*/*z* 193.0861 and 193.2396, while Panels **E**,**F** show EICs for *m*/*z* 177.0889 and 177.2387. In both ion pairs, the secondary ions (**D**,**F**) exhibit nearly identical retention times and peak shapes to their primary counterparts (**C**,**E**), suggesting a potential analytical relationship. This pattern parallels the observations from the urine specimen in Figure 5.

Regarding the feature 193.24315_0.872, neither MS-DIAL nor MS-FINDER suggests any candidate molecules. This feature has an almost identical RT to that of the feature 193.24315_0.872 (3-hydroxycotinine) but exhibits a slightly higher *m*/*z* (Figure 5A,C,D). It is also grouped together with the features of nicotine metabolites, including 3-hydroxycotinine (193.10092_0.876) in the low slicing metabolites (Terminal Branch Cluster C2-2) in Cluster C (Table 2C). Furthermore, the injection of authentic 3-hydroxycotinine yields both *m*/*z* 193.0861 and *m*/*z* 193.2396 at RT 0.90 min (Figure 6A,C,D). On the basis of these facts, we speculate that this higher-mass feature, 193.24315_0.872, would be derived from 3-hydroxycotinine, rather than being an unrelated artefact, even though there is no plausible explanation for this possible mass shift of ~0.14 Da.

### 3.13. Feature 179.12364_0.927

This feature likely corresponds to the molecular formula of C_10_H_14_N_2_O ([M + H]+ 179.117889). Possible chemical structures for C_10_H_14_N_2_O elucidated by MS-FINDER include 2-hydroxynicotine and nicotine-N-oxide (Figure 3). Among these metabolites, nicotine-N-oxide has been known as a minor primary metabolite of nicotine excreted in urine, whereas 2-hydroxynicotine has not been identified in human urine. The RT of nicotine-N-oxide is experimentally determined to be 0.96 min. Thus, this feature is identified as nicotine-N-oxide.

### 3.14. Features 177.10405_0.988 and 177.23868_0.976

These features likely correspond to the molecular formula of C_10_H_12_N_2_O ([M + H]+ 177.102239). Possible chemical structures for C_10_H_12_N_2_O elucidated by MS-FINDER include cotinine, the dominant nicotine metabolite in the urine (Figure 3) [53]. The RT is experimentally determined to be 1.0 min; thus, the feature 177.10405_0.988 is cotinine.

Regarding the feature 177.23868_0.976, neither MS-DIAL nor MS-FINDER suggests any candidate molecules. This feature has an almost identical RT to that of the feature 177.10405_0.988 (cotinine) but exhibits a slightly higher *m*/*z* (Figure 5B,E,F). It is also grouped together with the features of nicotine metabolites, including 3-hydroxycotinine (193.10092_0.876) and cotinine (177.10405_0.988) in the low slicing metabolites (Terminal Branch Cluster C2-2) in Cluster C (Table 2C). Furthermore, the injection of authentic cotinine yields both *m*/*z* 177.0889 and *m*/*z* 177.2387 at RT 1.05 min (Figure 6). On the basis of these facts, we speculate that this higher-mass feature, 177.23868_0.976, would be derived from cotinine, similar to the feature 193.24315_0.872 for 3-hydroxycotinine (see above). This phenomenon appears to be characteristic of cotinine and 3-hydroxycotinine, even though there is no plausible explanation for this possible mass shift of ~0.14 Da.

## 4. Discussion

This retrospective untargeted metabolomics analysis of routine UCDS data revealed the features significantly associated with cocaine exposure. Table 3 summarizes the annotated features, including known cocaine metabolites and related impurities, nicotine metabolites, an opioid metabolite (norfentanyl), and a tryptophan/serotonin metabolite (3-hydroxy-tryptophan).

Various features were annotated as known or putative cocaine metabolites, consistent with the expected urinary excretion patterns in cocaine users. Notably, one significant feature is likely ethyl ecgonine. Cocaine and alcohol are frequently used together by cocaine users [54,55,56]. While ethyl ecgonine has been previously reported in the context of ethanol and cocaine co-exposure [8], it has not been widely utilized as a biomarker of co-ingestion compared with cocaethylene, a toxic cocaine metabolite formed in the presence of ethanol [57]. However, the corresponding feature 318.17764_5.403 for cocaethylene (RT 5.55 experimentally) was filtered out by MetaboAnalyst during preprocessing as a non-informative feature due to low prevalence and signal intensity across most specimens. Our data indicate ethyl ecgonine as a valuable biomarker of ethanol and cocaine co-exposure.

In addition to cocaine-related compounds, other classes of exogenous metabolites were also annotated among the features significantly associated with cocaine-EIA. For example, co-use of tobacco products was evident in the dataset, with features corresponding to hydroxycotinine, cotinine, nicotine-N-oxide, and related metabolites. This aligns with known behavioral co-use patterns [58]. Additionally, norfentanyl, a major fentanyl metabolite, was also included among the significant features. Recent studies indicate both cross-contamination of fentanyl within illicit cocaine products and intentional co-use of cocaine and fentanyl, consistent with this finding [46,47,59,60,61,62,63,64].

Besides exogenous metabolites, the detection of 5-hydroxy-tryptophan as a discriminating feature between cocaine-EIA-positive and cocaine-EIA-negative groups is noteworthy. Cocaine alters tryptophan and serotonin metabolism [51]. Plasma serotonin levels are elevated among abstinent cocaine users, and psychiatric comorbidity among these users is associated with higher plasma serotonin levels compared with abstinent cocaine users without psychiatric comorbidity [52]. As 5-hydroxy-tryptophan is an immediate precursor of serotonin, it would be worth investigating if urinary 5-hydroxy-tryptophan levels could be used for stratification of cocaine-addicted patients for the risk of psychiatric symptoms during abstinence from cocaine.

This study has several limitations inherent to untargeted metabolomics workflows by LC-HRMS. The first issue is annotation uncertainty, an intrinsic limitation to metabolomics research [65,66], caused by the limited availability of authentic standards for most metabolites and the presence of isomeric molecules with shared molecular formulas. These isomeric molecules often yield similar MS/MS fragmentation results due to conserved substructures, complicating confident differentiation. For example, the molecular formula of C_9_H_13_NO_2_ ([M + H]+ 168.1019) can be for exogenous metabolites (e.g., cocaine metabolites (ecgonidine or methyl norecgonidine) and p/m-synephrine) or endogenous metabolites (e.g., 3-methoxytyramine), and these molecules have similar MS/MS spectra and RTs. Indeed, synephrine was identified along with coca alkaloids in Mariani wine, a popular 19th-century tonic wine with coca leaf extracts, by LC-high resolution-MS [67], but an alternative annotation of that feature might be either ecgonidine or the ISF of ecgonine. Even targeted data acquisition with the multiple reaction monitoring mode might lead to misidentification of isomeric molecules in LC-MS/MS analysis [68,69]. Authentic chemicals, different separation techniques (e.g., different LC conditions, ion mobility-MS), or confirmatory NMR analyses are required to overcome this challenge in LC-HRMS-based metabolomics. But these validation processes are not available to us. Our LC-qToF-MS instruments are heavily used for daily clinical toxicology testing, and we cannot use them under different LC settings just for investigational work.

Potential analytical artifacts are other issues. In-source fragmentation can produce artifactual features, whereas peak tailing can slightly shift RT values. Both phenomena can lead to false annotation of the features [15,70]. Furthermore, our MS dataset was centroided without lock mass correction, contributing to reduced mass accuracy and potential feature duplication. This was why we adopted rather generous mass tolerance parameters in MS-DIAL and MS-FINDER.

Other limitations include potential bias in the collected data and data preprocessing. We acquired MS data in positive ESI mode, which largely detects basic and neutral xenobiotics. However, the positive ESI mode might not be ideal for analyses of acidic metabolites, as they typically ionize better in negative ESI mode [71,72]. Thus, our dataset might not provide comprehensive coverage of acidic metabolites.

Sample preparation and normalization strategy can potentially cause bias. The mass spectrometry dataset was acquired using a dilute-and-shoot approach (1:4 dilution) of urine, which avoids analyte loss and facilitates broad metabolome coverage. However, this approach is also susceptible to matrix effects and ion suppression, especially in complex biological matrices like urine [14]. Although positive ESI mode is generally less prone to ion suppression than negative ESI mode when analyzing urine specimens for drug testing [73], matrix-related variability may still affect features. Additionally, we applied PQN to mitigate variability in urine concentrations across specimens [23], but no urinary creatinine-based normalization was performed. As a result, residual bias due to differences in hydration status may persist despite normalization efforts. Thus, the peak heights might not accurately reflect the amounts of the analytes in some specimens.

Despite these limitations, our study underscores the translational potential of repurposing clinical toxicology data generated by LC-qTof-MS for metabolomics research, offering a feasible approach to identifying clinically relevant but often overlooked biomarkers of drug exposure and drug-related metabolic disturbances in real-world settings.

## 5. Conclusions

This study demonstrates the utility of repurposing routine LC-qToF-MS data from clinical toxicology workflows for untargeted metabolomics analyses. By retrospectively analyzing UCDS data, we identified the 37 features most significantly associated with cocaine exposure, including known cocaine metabolites, potential indicators of co-exposures (e.g., ethyl ecgonine, norfentanyl, and nicotine metabolites), and alterations in endogenous metabolic pathways such as tryptophan metabolism. While many of these markers are not entirely novel, they remain underutilized in clinical and forensic toxicology, underscoring the value of untargeted metabolomics in expanding our understanding of cocaine-related metabolic disturbances. Our findings highlight the utility of routine MS data for discovering exposure biomarkers and improving knowledge of drug-induced metabolic changes in real-world populations.

Our study also highlights several technical and analytical challenges inherent in untargeted metabolomics using dilute-and-shoot workflows for urine specimens by LC-HRMS. These challenges include annotation uncertainty, analytical artifacts like in-source fragmentation, and potential biases arising from matrix effects, hydration status, and incomplete coverage of acidic metabolites due to the reliance on positive ESI mode. Further research is needed to address these technical and analytical gaps.

Future work should focus on validating the identified biomarkers using authentic standards, employing complementary analytical platforms, and expanding coverage to include the negative ESI mode. These efforts will be crucial in refining the biological interpretation of these features and assessing their clinical relevance in a broader toxicological context.

## Figures and Tables

**Table 1:** The patient demographics of this study. The number of cocaine-EIA-positive cases are given in parentheses.

	Male		Female	
Age (Years)	Outpatient	Non-Outpatient	Outpatient	Non-Outpatient
**0–9**	0	38 (2)	0	33
**10–19**	1	23	3	29
**20–29**	7	10 (1)	10	7 (3)
**30–39**	12 (4)	11 (2)	19 (3)	10 (1)
**40–49**	23 (4)	8 (2)	21 (2)	6 (3)
**50–59**	20	13 (1)	15	6 (1)
**60–69**	12 (1)	3 (2)	6	5
**70-**	5 (2)	4	2	2

**Table 2:** The 262 features associated with cocaine exposure grouped into seven high-level clusters, designated as Cluster A through Cluster G (corresponding to Table 2A–G), on the basis of hierarchical clustering of the normal intensity profiles. Within each high-level cluster table, data are grouped into sub-clusters (middle-level dendrogram slices) (e.g., A1, A2), which are represented as columns of the table. Within each sub-cluster or column, terminal branches (low-level dendrogram slices) are grouped and labeled (e.g., A1-1, A1-2, A1-3). This hierarchical clustering framework was used to capture co-varying features, which may include parent compounds, metabolites, and related ion species.

**Cluster A (Table 2A)**												**Cluster B (Table 2B)**			
A1			A2					A3				B1			
A1-1			A2-1					A3-1				B1-1			
90.05575_0.743			145.13571_1.285					154.12878_1.735				90.97554_0.714			
241.12184_0.698			187.14908_1.289					155.13155_1.637				106.95014_0.713			
A1-2			206.08679_2.052					170.12999_1.403				158.96283_0.713			
142.12558_0.854			304.16803_4.254					361.21246_1.4				174.93845_0.715			
201.16602_1.997			328.18445_1.433					A3-2				175.05_0.912			
400.20502_8.74			330.17252_5.513					236.13498_4.346				216.92113_0.717			
A1-3			332.24408_3.986					317.20694_1.113				226.95103_0.713			
330.41934_5.048			346.2359_4.045					317.21439_1.06				234.89557_0.719			
402.28555_7.96			560.30536_5.815									242.92693_0.715			
716.56219_12.134			A2-2									244.92462_0.724			
960.61224_5.519			207.11478_4.599									272.94611_0.7			
			260.22177_5.457									294.93652_0.718			
			290.14951_3.314									310.91443_0.726			
			304.20865_2.685									316.87534_0.712			
			306.13705_6.42									352.89661_0.718			
			332.24271_4.252									420.88394_0.717			
			339.97421_0.665									430.91025_0.712			
			358.25793_6.353									446.88776_0.717			
			359.22852_4.294									452.84384_0.716			
			370.22571_4.493									B1-2			
			370.23047_4.832									98.92009_0.73			
			398.26291_6.445									172.04063_0.842			
			444.31995_6.079									200.0448_0.702			
			460.22427_4.308									210.93623_0.718			
			546.26807_6.705									218.92108_0.718			
			A2-3									232.91742_0.718			
			257.15521_1.881									268.03149_0.717			
			358.44699_8.378									336.01816_0.71			
			358.60458_8.576									378.90033_0.719			
			398.25073_6.293												
			A2-4												
			479.24384_3.894												
			481.25757_4.358												
			529.2973_4.732												
			553.29901_9.038												
Cluster C (Table 2C)							Cluster D (Table 2D)								
C1		C2					D1			D2					D3
C1-1		C2-1					D1-1			D2-1					D3-1
91.05293_0.83		135.05078_2.818					91.05488_2.605			94.03997_0.693					116.22058_0.802
168.11021_0.847		230.1496_1.057					149.06212_3.642			105.03697_0.875					200.09056_1.02
200.12935_0.795		230.29869_1.051					186.01846_4.664			290.10568_6.327					200.09056_1.294
441.17444_0.915		168.10132_1.248					D1-2			522.40491_12.074					D3-2
C1-2		168.10931_1.568					121.04194_0.825			D2-2					168.06374_1.28
117.05989_0.864		184.10083_1.021					122.07076_0.782			107.04818_1.115					186.08173_1.203
139.12474_1.347		186.11212_1.025					137.19272_0.733			546.40143_11.75					200.09158_0.796
163.12866_0.849		186.11491_0.834					150.05968_0.839			D2-3					
177.34515_0.976		192.10089_1.04					152.07004_2.343			131.11659_0.693					
198.11752_1.083		208.09537_1.018					191.02417_0.942			402.37381_11.955					
214.10306_1.408		C2-2					D1-3			534.41858_11.965					
216.12822_1.072		177.10405_0.988					140.07437_0.729			D2-4					
C1-3		177.14621_0.68					185.07774_1.451			172.10063_1.902					
150.09793_0.812		177.23868_0.976					201.08185_1.004			173.08467_2.303					
162.108_2.008		179.12364_0.927					265.11493_0.706			D2-5					
186.11717_1.818		193.10092_0.876					D1-4			187.06279_0.952					
258.08966_1.448		193.13478_0.697					155.07788_0.851			238.84851_0.721					
339.16333_0.701		193.24315_0.872					182.08345_0.819			256.82074_0.732					
		357.22891_0.88					268.09927_0.854			384.85068_0.716					
		C2-3								400.82483_0.724					
		214.15097_0.935													
		240.10555_1.076													
		264.12234_0.734													
		475.2739_9.493													
		504.31165_11.765													
		C2-4													
		218.08513_1.244													
		221.08717_1.055													
		282.15509_4.618													
		309.13354_1.511													
		331.22858_4.093													
		388.22858_3.758													
Cluster E (Table 2E)						Cluster F (Table 2F)								Cluster G (Table 2G)	
E1	E2					F1			F2					G1	
E1-1	E2-1					F1-1			F2-1					G1-1	
104.10567_0.733	104.10567_11.995					106.06516_1.994			153.13197_4.525					116.05332_11.649	
104.2101_0.734	104.10775_11.868					256.18127_1.385			311.15509_4.525					135.04501_11.694	
152.065_2.776	104.10775_11.447					258.08966_1.149			378.18292_2.252					311.34421_11.694	
207.15585_2.426	149.05659_1.326					300.20895_1.719			F2-2					312.16464_12.233	
233.17049_3.226	213.12489_0.836					330.18475_1.651			165.07965_0.797					312.16464_11.693	
E1-2	395.29956_11.649					338.26901_11.447			224.12656_0.967					527.40106_12.155	
125.06116_2.653	E2-2					498.27695_6.524			277.12286_2.952					593.33722_10.015	
223.11052_1.41	104.10567_0.978					554.4505_12.18			292.15628_2.97					659.48175_11.901	
323.14807_2.849	142.08922_1.821					680.44757_11.809			519.40143_12.072					G1-2	
382.83783_0.703	149.06207_4.254					F1-2			544.40845_11.628					142.12054_0.995	
449.16858_4.928	169.10295_0.889					124.07899_1.053			639.43219_12.067					203.06929_2.133	
470.35092_11.745	E2-3					253.05179_3.2			F2-3					237.07332_1.753	
E1-3	165.07159_4.713					278.14926_2.887			180.08086_2.536					303.16507_3.432	
462.8587_0.722	165.07463_5.316					301.11752_4.474			235.13065_0.829					G1-3	
467.10083_11.731	218.0378_1.446					329.21564_3.3			F2-4					146.05814_1.87	
	250.86957_0.719					332.25024_4.576			195.12511_1.716					188.06906_1.861	
	256.17471_1.742					183.09117_0.77			246.10471_0.754					247.14194_1.873	
	350.02979_0.703					201.16733_0.774			256.18103_6.388					247.29958_1.876	
	468.81296_0.725					237.131_1.815			F2-5					G1-4	
	498.89853_0.711					290.20178_3.419			239.15465_1.732					182.123_0.87	
						362.12997_4.903			283.18033_2.464					200.13109_4.253	
						F1-3			300.21008_1.962					200.13182_1.007	
						220.12494_1.151			344.23004_1.752					200.13376_1.911	
						220.27379_1.155								292.1264_4.051	
						242.0983_1.15								304.15652_4.399	
						247.09369_1.997									
						254.17285_0.906									
						261.15073_2.827									
						330.17572_5.651									
						354.23468_5.676									
						F1-4									
						493.37396_11.814									
						544.42969_11.838									
						553.40015_11.93									

**Table 3:** Thirty-seven significant features positively associated with cocaine-EIA results selected by at least two analyses.

Feature	Correlation	Volcano	PLS	EBAM	SAM	RF	Annotation
200.12935_0.795	X	X				X	Methyl ecgonine *
304.15652_4.399	X	X	X	X	X	X	Cocaine *
200.13182_1.007	X	X				X	Ethyl norecgonine (putative)
182.123_0.87	X	X	X	X	X	X	ISF (-H2O) of methyl ecgonine
186.11491_0.834	X	X	X	X	X	X	Ecgonine *
330.17252_5.513	X	X				X	Cinnamoylcocaine (putative)
168.11021_0.847	X	X	X	X	X	X	Ecgonidine *#
198.11752_1.083	X	X					Unknown
277.12286_2.952	X	X				X	Unknown
330.17572_5.651	X	X				X	Cinnamoylcocaine (putative)
162.108_2.008	X	X				X	Unknown
186.11212_1.025	X	X	X	X	X	X	Methyl norecgonine (putative)
214.15097_0.935	X	X					Ethyl ecgonine *
233.17049_3.226	X	X					Norfentanyl *
186.11717_1.818	X	X				X	ISF of Hydroxy-benzoylecgonine (putative)
200.13109_4.253	X	X				X	Unknown $
91.05293_0.83	X	X					Unknown
292.1264_4.051	X	X					N-Hydroxy-norbenzoylecgonine (putative)
168.10931_1.568	X					X	Methyl norecgonidine (putative)
290.14951_3.314	X	X					Benzoylecgonine ^
216.12822_1.072	X	X					Unknown
168.10132_1.248	X	X		X	X		Methyl norecgonidine and ISF of methyl norecgonine # (putative)
304.16803_4.254		X	X	X	X		Cocaine *
155.13155_1.637		X		X			Unknown
560.30536_5.815		X		X			Unknown
221.08717_1.055		X	X	X			5-Hydroxy-L-tryptophan *
193.13478_0.697		X		X			Unknown
206.08679_2.052		X	X				Unknown
292.15628_2.97		X	X				Unknown
104.2101_0.734		X	X				Unknown
188.06906_1.861		X	X				Unknown
193.10092_0.876				X	X	X	3-Hydroxycotinine #
177.23868_0.976				X	X	X	Cotinine artifact (putative)
177.14621_0.68				X	X	X	Unknown
177.10405_0.988				X	X		Cotinine *
179.12364_0.927				X		X	Nicotine-N-oxide *
193.24315_0.872				X		X	3-Hydroxycotinine artifact (putative)

* Confirmed by spike study. ^ Tail part. # A composite peak by more than one analyte ion cannot be ruled out. $ A cocaine metabolite or its ISF is suspected.

**Table 4:** Retention times (RTs) of cocaine-related metabolites, nicotine-related metabolites, other xenobiotics, and endogenous metabolites in urine, as determined experimentally and predicted using various computational models. The predicted RTs were calculated using the R package Retip based on the following models: XGBoost (xgb), Random Forest (rf), Bayesian Neural Network (brnn), and an automatic machine learning tool using the following molecular descriptors: logP (XLogP), atomistic logP (ALogP), number of hydrogen bond donors (nHBDon), and number of basic groups (nBase).

	RT (Experimentally Determined)	xgb	Rf	brnn	XLogP	ALogP	nHBDon	nBase
**Cocaine-related metabolites**								
Benzoylecgonine	2.95	3.1	3.36	2.56	3.18	3.02	3.17	3.2
c*is*-Cinnamoylcocaine		6.94	7.02	4.77	6.38	5.99	5.93	5.99
tr*ans*-Cinnamoylcocaine		6.94	7.02	4.77	6.38	5.99	5.93	5.99
Cocaethylene	5.55	5.79	5.58	5.39	5.56	5.63	5.16	5.84
Cocaine	4.46	4.26	4.32	4.11	4.37	4.61	4.52	4.53
Ecgonidine	0.84	1.24	1.73	1.05	1.18	0.91	1.69	0.91
Ecgonine	0.80	0.51	1.3	0.34	0.29	0.87	1.27	1.52
Ethyl ecgonine	0.93	1.95	3.05	1.85	2	1.9	1.79	1.9
Ethyl norecgonidine		3.08	3.46	3.52	2.16	2.03	1.66	2.03
Ethyl norecgonine		1.84	2.93	2.18	1.83	1.58	1.56	1.58
Methyl ecgonidine	1.10	1.39	1.69	1.62	1.32	1.16	1.5	1.71
Methyl ecgonine	0.80	0.88	1.19	1	0.7	0.74	0.91	0.83
Methyl norecgonidine		2.76	3.17	2.4	1.55	1.59	1.05	1.59
Methyl norecgonine		0.87	1.32	1.25	1	1.17	1.1	1.17
m-Hydroxy-benzoylecgonine		2.68	3.67	2.17	3.89	4.14	3.77	4.14
o-Hydroxy-benzoylecgonine		2.94	4	2.12	2.97	3.28	2.63	3.28
p-Hydroxy-benzoylecgonine		2.74	3.67	1.96	3.86	4	3.77	4
*m*-Hydroxy-benzoylnorecgonine		2.66	3.11	2.6	2.45	2.44	2.63	3.23
N-Hydroxy-benzoylnorecgonine		3.22	3.15	4.12	2.84	2.74	3.08	3.18
*o*-Hydroxy-benzoylnorecgonine		2.81	3.61	2.82	2.84	2.79	3.29	3.84
*p*-Hydroxy-benzoylnorecgonine		2.66	3.1	2.41	2.46	2.38	2.63	3.23
Norcocaine	4.54	4.32	4.05	4.47	4.1	4.32	4.19	4.31
**Nicotine-related metabolites**								
Cotinine N-oxide		1.18	1.41	0.76	1.59	1.65	1.62	1.29
Cotinine	1.06	1.12	1.23	0.65	1.18	1.2	1.38	1.39
2-Hydroxynicotine		0.7	1.4	-0.18	0.55	0.8	1.14	0.79
3-Hydroxycotinine	0.90	0.78	1.36	0.21	0.86	0.96	1.23	1.01
5-Hydroxycotinine		1.11	1.37	-0.03	1.39	1.07	1.78	2.05
Nicotine N-oxide	0.92	1.07	1.4	0.12	1.2	1.29	1.28	1.42
**Other xenobiotics**								
*p*-Synephrine	0.83	0.92	1.5	0.55	0.89	0.81	1.35	1.21
Phenylephrine (*m*-synephrine)	0.83	0.92	1.47	0.62	0.81	0.83	1.31	1.06
Norfentanyl	3.25	3.24	3.49	2.78	3.32	3.26	3.42	3.16
**Endogenous metabolite**								
3-Methoxytyramine	0.98	1.5	1.53	1.94	1.19	1.18	1.17	1.43
5-Hydroxytryptophan	0.90	1.19	1.66	0.25	0.78	0.71	1.6	1.34

## Data Availability

The datasets presented in this article are not readily available because the data are part of an ongoing study. The original datasets generated and/or analyzed during the current study contain protected health information (PHI) and thus cannot be publicly shared due to the Health Insurance Portability and Accountability Act (HIPAA) Privacy Rule in the US.

## Abbreviations

The following abbreviations are used in this manuscript.

MS	Mass spectrometry
LC-qToF-MS	Liquid chromatography–quadrupole time-of-flight mass spectrometry
EIA	Enzyme immunoassay
LC-HRMS	Liquid chromatography–high-resolution mass spectrometry
UCDS	Urine comprehensive drug screening
UPMC	University of Pittsburgh Medical Center
ESI	Electron spray ionization
PQN	Probabilistic quotient normalization
SAM	Significance analysis of microarrays and metabolites
EBAM	Empirical Bayesian analysis of microarrays and metabolites
FDR	False discovery rate
PLS-DA	Partial least squares discriminant analysis
RT	Retention time
xgb	XGBoost
rf	Random Forest
brnn	Bayesian Neural Network
